# The Remarkable Effect of Potassium Iodide in Eosin and Rose Bengal Photodynamic Action against *Salmonella* Typhimurium and *Staphylococcus aureus*

**DOI:** 10.3390/antibiotics8040211

**Published:** 2019-11-05

**Authors:** Adriele R. Santos, Andréia F. P. Batista, Ana T. P. C. Gomes, Maria da Graça P. M. S. Neves, Maria Amparo F. Faustino, Adelaide Almeida, Noboru Hioka, Jane M. G. Mikcha

**Affiliations:** 1 Postgraduate Program in Food Science, State University of Maringá, Maringá 87020-900, Brazil; andreia.farias04@hotmail.com; 2 Department of Biology and CESAM, University of Aveiro, 3810-193 Aveiro, Portugal; ana.peixoto@ua.pt; 3 QOPNA& LAQV-REQUIMTE and Department of Chemistry, University of Aveiro, 3810-193 Aveiro, Portugal; gneves@ua.pt (M.d.G.P.M.S.N.); faustino@ua.pt (M.A.F.F.); 4 Department of Chemistry, State University of Maringá, Maringá 87020-900, Brazil; nhioka@uem.br; 5 Department of Clinical Analysis and Biomedicine, State University of Maringá, Maringá 87020-900, Brazil

**Keywords:** xanthene derivatives, photodynamic inactivation, inorganic salt, antimicrobial resistance, *Salmonella*

## Abstract

Antimicrobial photodynamic therapy (aPDT) has been shown as a promising technique to inactivate foodborne bacteria, without inducing the development of bacterial resistance. Knowing that addition of inorganic salts, such as potassium iodide (KI), can modulate the photodynamic action of the photosensitizer (PS), we report in this study the antimicrobial effect of eosin (EOS) and rose bengal (RB) combined with KI against *Salmonella enterica* serovar Typhimurium and *Staphylococcus aureus*. Additionally, the possible development of bacterial resistance after this combined aPDT protocol was evaluated. The combination of EOS or RB, at all tested concentrations, with KI at 100 mM, was able to efficiently inactivate *S*. Typhimurium and *S. aureus*. This combined approach allows a reduction in the PS concentration up to 1000 times, even against one of the most common foodborne pathogenics, *S*. Typhimurium, a gram-negative bacterium which is not so prone to inactivation with xanthene dyes when used alone. The photoinactivation of *S*. Typhimurium and *S. aureus* by both xanthenes with KI did not induce the development of resistance. The low price of the xanthene dyes, the non-toxic nature of KI, and the possibility of reducing the PS concentration show that this technology has potential to be easily transposed to the food industry.

## 1. Introduction

The access to safe food is considered as an important requirement to guarantee the quality of human life in modern society [1,2]. In fact, outbreaks of foodborne diseases are one of the main causes of morbidity and mortality being considered as an international public health problem [3], causing significant social and economic impacts [4]. According to the World Health Organization (WHO), it is estimated that more than 600 million people get sick as the result of unsafe food consumption [5,6]. One of the emerging problems related with foodborne bacteria is the increase of antibiotic resistance. It is known that changes in the patterns of food consumption (the preference for fresh and minimally processed foods), alterations in the globalization of the food market, and the emergence of multidrug resistant (MDR) bacteria have turned the control of foodborne diseases into a challenge [7,8]. According to the Centre for Disease Control and Prevention [9], about 400,000 people per year are affected by foodborne infections caused by MDR bacteria in the United States. Multidrug resistant *Salmonella* spp. and *Staphylococcus aureus* are a cause of concern since they have been isolated from meat, poultry, and dairy [10,11,12,13,14].

Nowadays, it is assumed that the development of novel antibiotics will not solve the MDR bacteria problem, since microorganisms may find new pathways of resistance to these new molecules. Therefore, efforts should be made towards the development of more efficient, non-toxic, and noninvasive antimicrobial methods to apply to the hosts. Importantly, these new methods should not induce the development of antimicrobial resistance [15,16,17]. Toward this end, antimicrobial photodynamic therapy (aPDT) has been considered as a promising non-antibiotic approach to inactivate foodborne bacteria [18,19,20,21,22,23].

aPDT involves the use of a photosensitizer (PS) that when excited by light reacts with molecular oxygen producing reactive oxygen species (ROS) such as singlet oxygen and/or hydroxyl radicals, superoxide, and hydrogen peroxide [15,23]. These ROS can react with biological molecules (e.g., proteins, lipids, and nucleic acids) causing microbial death [16,24,25]. This technique presents several advantages when compared with the use of traditional antimicrobials, showing to be efficient independently of the antimicrobial resistance profile and to prevent further development of resistance even after several cycles of treatment [15,16,17,26]. This approach has been efficient to inactivate several microorganisms, such as gram-negative and gram-positive bacteria [18,19,21], fungi [15,27,28,29], and viruses [15,30], and to degrade the matrix of microbial biofilms and kill the resident bacteria [16,31,32].

**Figure 1:**
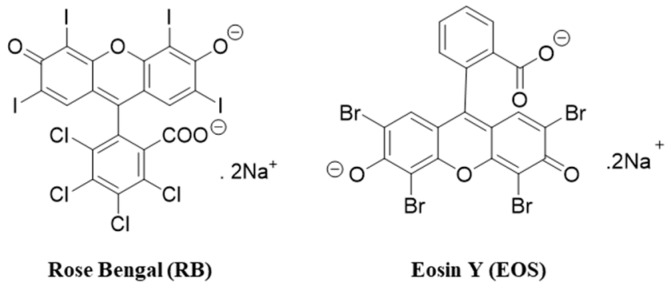
Chemical structures of rose bengal (RB) and eosin Y (EOS).

An ideal PS is a molecule that is present, in general, with a high quantum yield of singlet oxygen (Φ_Δ_), low photo-bleaching yield, high affinity for the targeted site, and high stability [33,34]. Xanthene dyes have been considered good PSs to induce bacterial photoinactivation due to their low price, high molar absorptivity, and high singlet oxygen quantum yield (Φ_Δ_) [18,33,35]. The xanthene dyes, rose bengal (RB) and eosin Y (EOS) (Figure 1), have already proven to be effective against gram-positive and gram-negative bacteria [19,20,31,36], however, these dyes showed to be more effective against gram-positive bacteria. This limitation can be overcome by the use of different organic salts such as sodium bromide, sodium azide, sodium thiocyanate, and potassium iodide (KI) [36,37,38]. Recently, some studies have demonstrated that the combinations of PSs and the inorganic salt KI improve the efficiency of aPDT [15,36,39,40,41]. Some xanthene dyes are approved for use in drug, cosmetic, and medical applications, and as food additives [19,31], while the safety of KI has been reported by a Food and Drug Administration (FDA) document [42].

aPDT certainly is a promising tool to inactivate food and food surfaces. However, to adopt and implement photoinactivation in the food industry, a variety of factors, both those related to aPDT and those related to the food matrix, need to be evaluated [3,43]. Most of the studies with aPDT in food matrices or food-related contamination have been done at laboratory scale, and have focused on fruits, vegetables, and poultry, or food contact surfaces [3,43]. Tao et al. [22] applied different concentrations of curcumin in fresh-cut Fuji apple inoculated with *Escherichia coli*. The fruits were illuminated with a 420 nm LEDs on both sides, at a 4 cm distance from the LED. The authors observed a reduction in *E. coli* population, as well in the activity of the enzymes polyphenol oxidase and peroxidase. Aurum and Nguyen [44] achieved a 2 log inactivation of *E. coli* on grapes treated with curcumin at 1.6 μM. The grapes were immersed in curcumin solutions containing the inoculum for 60 min and the samples were afterwards irradiated with a blue LED light (465–470 nm). Luksiene and Paskeviciute [45], using Na-Chl at 0.75 μM and a 405 nm LED, tested the efficacy of PDI against *Listeria monocytogenes* Ly 56 cells attached on polyolefine. They observed that the aPDI were able to eliminate a 4 log CFU/cm^2^ of bacterial population. The results of these studies showed that no negative effects were observed in the food matrices [22,44].

Therefore, the aim of this work was to investigate the antimicrobial photodynamic effect of the xanthene dyes RB and EOS combined with the inorganic salt KI against *Salmonella* Typhimurium and *Staphylococcus aureus*. Additionally, the aim was to evaluate the bacterial resistance induced by the combination of RB/EOS, KI, and light irradiation.

## 2. Results and Discussion

### 2.1. Photostability Assay

**Figure 2:**
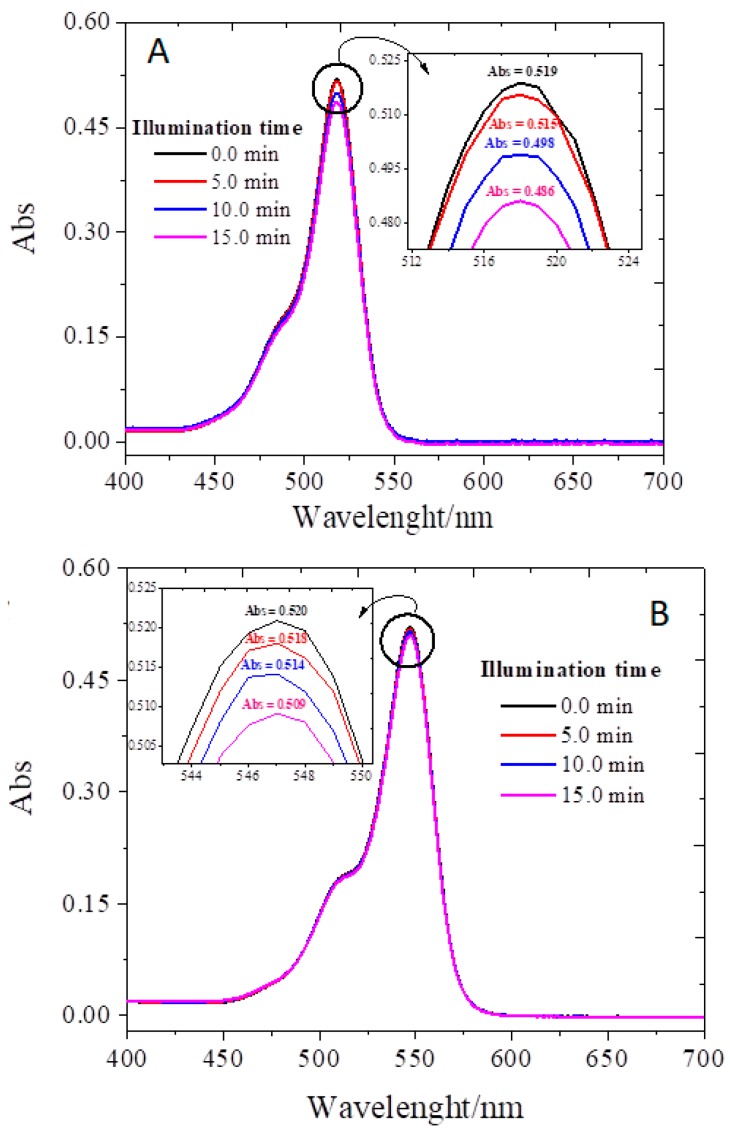
Photobleaching of EOS (**A**) and RB (**B**) without KI in PBS illuminated by a set of LEDs (10 mW/cm^2^ and a wavelength of 530 ± 40 nm) for a period of 0, 5, 10, and 15 min.

The absorption spectra of EOS (Figure 2A) and RB (Figure 2B) before and after being irradiated for 15 min, under the conditions used in the photodynamic assays (vide infra) show a slight decrease in the maximum absorption intensity (ca 6% and 2%). The decrease is dependent on the irradiation time and these results are in agreement with the work of Rabello et al. [46], where it was reported that EOS has a higher tendency to suffer photobleaching than RB. In future research the absorption spectra of the combined use of xanthenos dyes with KI may be conducted to better ascertain the use of these PSs in association with green LED light to control bacterial contamination.

### 2.2. Photodynamic Inactivation Assays

**Figure 3:**
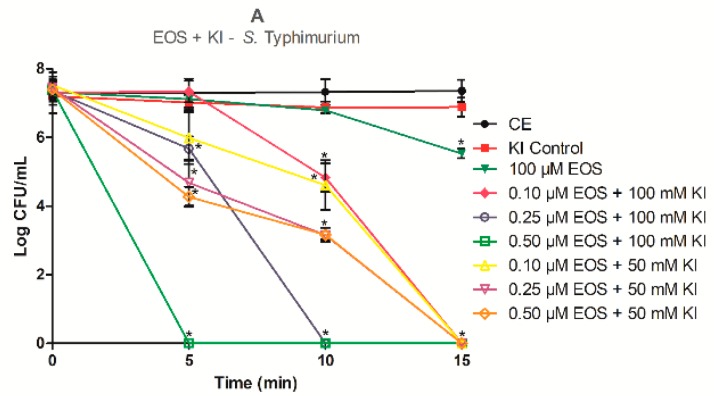

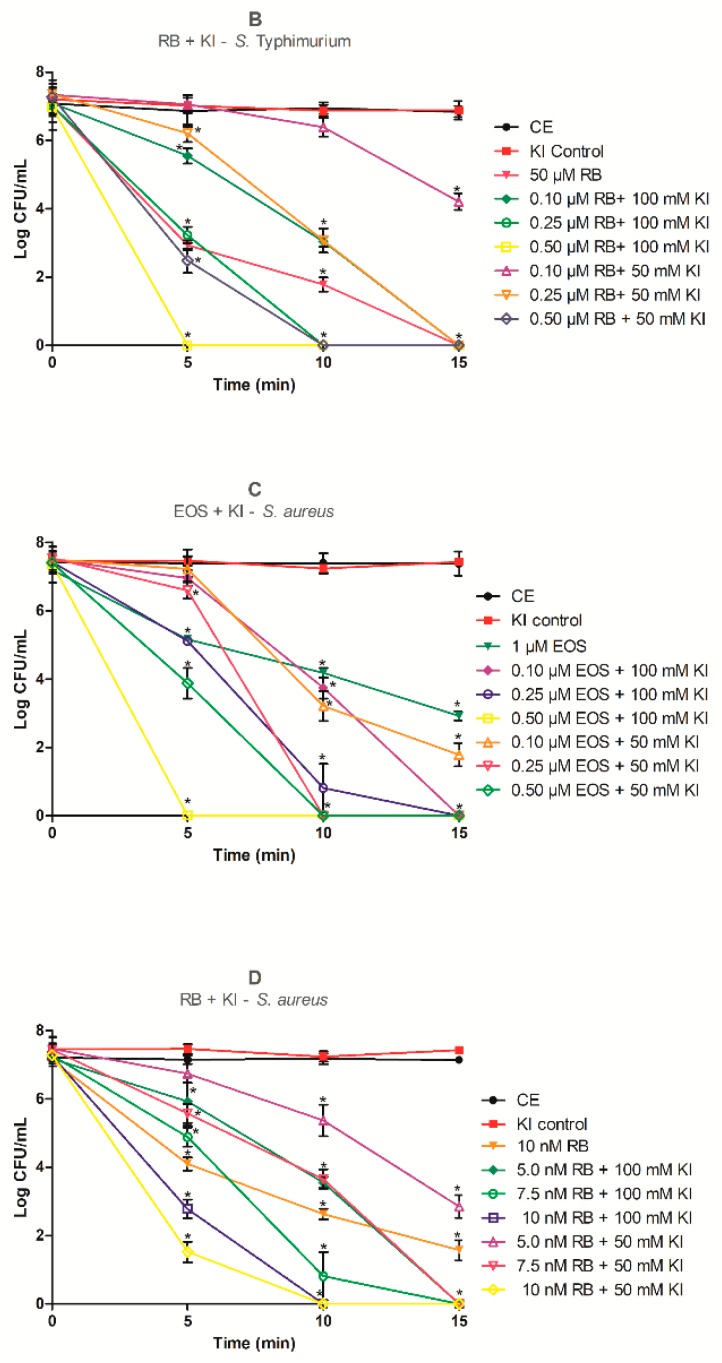
Effect of different times of irradiation and concentrations of EOS and RB combined with KI in the inactivation of *Salmonella* Typhimurium (**A**,**B**) and *Staphylococcus aureus* (**C**,**D**) cells. Samples were incubated in the dark for 10 min and then subjected to 5, 10, or 15 min of green (530 ± 40 nm) LED light exposure. The control group represents the cells in phosphate-buffered saline (PBS). Data are presented as mean values and the error bars indicate the standard deviation. * *p* < 0.05. Lines just combine the experimental points.

Although the xanthene derivatives dyes RB and EOS, in aqueous buffer solution, show high singlet oxygen quantum yield (Φ_Δ_ = 0.75 for RB, and Φ_Δ_ = 0.57 for EOS), which is enough to inactivate gram-positive bacteria, at neutral pH, they are dianionic protolytic molecules [33]. This is a limitation for the photoinactivation of gram-negative bacteria, once they are mostly impermeable to anionic or neutral charged dyes [41]. However, recently Hamblin described an efficient photoinactivation of *E. coli* in the presence of an anionic porphyrin combined with KI [37]. Having this mind, we have decided to study if the photodynamic effect of EOS and RB towards *S. aureus* and *S*. Typhimurium is potentiated by KI. The concentration of RB, EOS, and KI, as well as the irradiation times were chosen based on previous studies of our research group [15,19,31]. So, RB was tested at 10.0 nM alone and, at 5.0 nM, 7.5 nM, and 10 nM combined with KI, for *S. aureus* (a gram-positive bacterium) and at 50 µM alone and, 0.10 µM, 0.25 µM, and 0.50 µM with KI, for *S*. Typhimurium (a gram-negative bacterium). The concentrations of EOS for the assays in the absence of KI were 1.0 µM for *S. aureus* and 100 µM for *S*. Typhimurium. In the presence of KI, the EOS concentrations tested were 0.10 µM, 0.25 µM, and 0.50 µM for both *S. aureus* and *S*. Typhimurium. In these assays the KI concentrations used were of 50 mM and 100 mM for both bacteria. The results obtained are presented in Figure 3. The dark control samples (PSs + KI in the dark (DC)) and the light control (bacteria strains only irradiated with LED (LC)) (data not shown) had no reductions on bacterial population compared with bacterial control group (bacterium strain only in PBS). The KI control also did not show differences in the *S. aureus* and *S*. Typhimurium cells reduction when compared with the control group (*p* < 0.05), as shown in Figure 3A–D.

The results obtained for the inactivation of *S*. Typhimurium mediated by EOS and RB alone show that these PSs have a limited efficacy in the photoinactivation of this gram-negative bacterium (Figure 3A,B). When EOS was used alone (Figure 3A), even at 100 µM and after an irradiation period of 15 min (light dose of 9.0 J/cm^2^), the reduction in the survival of *S*. Typhimurium cells was only about 2 log (*p* < 0.05). Bonin et al. [19] has shown that EOS irradiated for 15 min with green light (530 ± 40 nm) promoted a slight reduction of about 1 log in *S*. Typhimurium survival using the EOS at 10 µM. These results show that increasing the concentration of EOS led to different photoinactivation profiles of *S*. Typhimurium cells. When RB alone was used (Figure 3B) it was possible to observe the total inactivation of *S*. Typhimurium cells with a concentration of 50 µM and 15 min of irradiation (9.0 J/cm^2^). Silva et al. [31] also achieved the complete inactivation of *S*. Typhimurium cells with a small irradiation time (5 min.), but they used RB at 75 µM. So, even reaching the inactivation of *S*. Typhimurium cells until the detection limit of the method with RB, it was necessary for a high concentration of the PS, and this could be a barrier to its application in the food industry.

The combined effect of EOS and RB with KI against *S*. Typhimurium (Figure 3A,B) show that this combination is effective in the photoinactivation of this gram-negative bacterium. For the combination 0.10 µM EOS with 100 mM KI total inactivation was observed after 15 min of irradiation (light dose of 9.0 J/cm^2^), while for the combinations 0.25 µM EOS + 100 mM KI and 0.50 µM EOS + 100 mM KI the limit detection of the methodology was achieved after 10 min (6.0 J/cm^2^) and 5 min (3.0 J/cm^2^) of irradiation, respectively (*p* < 0.05). With the combination EOS + KI it was possible to completely inactivate the *S*. Typhimurium cells using a PS concentration 1000 times smaller. These data show that KI effectively potentiates EOS in aPDT against this gram-negative bacterium. For RB, our results also show an aPDT effect surprisingly high, promoting a reduction in the RB concentration up to 200 times against *S*. Typhimurium (Figure 3B). In this case it was possible to inactivate *S*. Typhimurium until the detection limit of the method for all combinations of RB with 100 mM KI as shown in Figure 3B (*p* < 0.05). With the concentration of RB 0.50 µM with 100 mM KI no culturable cells were recovered even after 5 min of light exposure (3.0 J/cm^2^). In agreement with previous studies [38,39], it was also possible to observe that the photoinactivation rate increases with the increase of the PS or KI concentration or with the time of irradiation (*p* < 0.05; Figure 3). Our results are in accordance with Wen et al. [41] and Vieira et al. [36] that showed a great improvement in the action of the xanthene dye RB against gram-negative bacteria with the addiction of KI.

The use of EOS and RB alone in aPDT treatment against *S. aureus* cells proves to be more effective than for *S*. Typhimurium (Figure 3C,D). Nevertheless, it was not possible to achieve complete inactivation of the bacterium cells, even with the longest irradiation time (15 min; 9.0 J/cm^2^). On the other hand, Bonin et al. [19] used 5.0 µM of EOS alone to achieve the total inhibition of the bacterium at 5 min of light exposure. While Silva et al. [20] reported that it was necessary to use 25 nM RB alone to achieve total inhibition of *S. aureus* cells with the same time of light exposure (5 min).

Additionally, the combination of EOS and RB with KI also achieved a good improvement in the photoinactivation action against *S. aureus* (Figure 3C,D). When experiments were performed with EOS at 0.10 µM and 0.25 µM with 100 mM KI a total inactivation was observed after 15 min (9.0 J/cm^2^) of irradiation. When the concentration of EOS was increased for 0.50 µM no cultivable cells were recovered after 5 min (3.0 J/cm^2^) of irradiation (*p* < 0.05; Figure 3C). Instead, for EOS alone at 1 µM and 5 min (3.0 J/cm^2^) of irradiation, a reduction of about only 2 logs was achieved. In the photoinactivation mediated by RB with 100 mM KI, the total inactivation of *S. aureus* was observed for all PS studied concentrations (*p* < 0.05; Figure 3D). When it was used RB at 10 nM with 100 mM KI the total inactivation of the bacterium cells was achieved with 10 min (6.0 J/cm^2^) of irradiation (*p* < 0.05). While when the RB was used alone, in the same concentration and time of irradiation, it was observed that it achieved a reduction of about 3 logs in the *S. aureus* cells (*p* < 0.05).

In our aPDT studies, namely when KI was used, the necessary PS concentration to inactivate the bacteria was very low, which probably would not affect the food. However, in a near future, further experiments, using food matrices, are needed in order to evaluate the potential of this combined aPDT approach in food industry.

It was possible to observe that the effect of KI was more pronounced when combined with EOS rather than RB, as expected. According to Huang et al. [47] when a PS already has a pronounced activity, such as RB, further improvements are more difficult to be achieved. But as EOS has a lower activity on its own, KI easily improved its photodynamic effect.

Some research groups that studied the use of PSs with KI in aPDT stated that, when a PS is used in combination with KI, it is necessary to have lower PS concentration or lower light exposure than when used in non-combined strategies [15,36,38,39,40,41,47,48,49]. The potentiated effect of RB by KI was studied in the photoinactivation of *E. coli* and *S. aureus* [36,41]. *E. coli* cells exposed to RB with KI and a light dose of 10 J/cm^2^ (540 ± 15 nm) were reduced in more than 6 logs, while when KI was omitted, less than 1 log of killing was found [41]. Vieira et al. [36] also demonstrated that RB alone showed no photoinactivation effect in *E. coli* cells, but that the addition of KI provided the reduction of the cells until the detection limit of the method was reached. Methicillin-resistant *Staphylococcus aureus* (MRSA) was reduced about 2 logs with 100 nM of RB alone plus light (20 J/cm^2^), but when KI (100 mM) was added eradication of cells at 20 J/cm^2^ was observed [41]. Importantly, to our knowledge, there are no reports of the combined use of EOS with KI against *S. aureus* and *S*. Typhimurium.

Some studies have shown that KI also enhances the effect of other PS classes [36,38,39,40,47,48,49]. The potentiated effect of KI was observed for porphyrin-based PSs, Photofrin [40] and, for a formulation constituted by five cationic porphyrin derivatives [15], in the photoinactivation of gram-negative and gram-positive bacteria. The combined effect of MB and KI for the photoinactivation of *E. coli* and *S. aureus* was also shown [36,39]. These authors observed that the addition of KI increased the bacterial killing in 4 logs for *S. aureus* and 2 logs for *E. coli* [39], as well as reduced the time of light exposure of 150 min to 30 min for *E. coli* inactivation [36]. So, when comparing our results with these results, we can say that our findings are in line with them.

**Figure 4:**
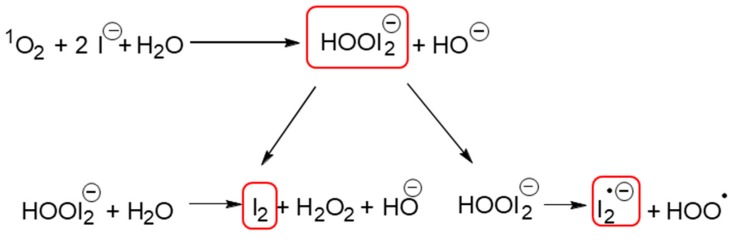
Schematic representation of the decomposition of peroxyiodide into free iodine (I_2_/I_3_^−^) and hydrogen peroxide (H_2_O_2_) or iodine radicals (I_2_^•−^) (elaborated according with the literature [24,37,38,39,40,43,44,45]).

All the aforementioned studies helped to elucidate how KI acts in the potentiation of aPDT. Huang et al. [40] proposed that, for porphyrins, the reaction mechanism occurred via singlet oxygen (^1^O_2_), once they observed an increase of the oxygen consumption when Photofrin was irradiated in the presence of KI, as well the generation of hydrogen peroxide. Rose bengal and EOS show high singlet oxygen quantum yield and they operate predominantly via the type II photochemical pathway, as well as porphyrins. So, for these dyes this extra killing effect of KI is caused by several parallel reactions that initiates with the reaction of ^1^O_2_ with KI [15,36,37,38,40,41,49] (Figure 4). These reactions could produce free iodine (I_2_/I_3_^−^) and hydrogen peroxide (H_2_O_2_), that are stable species, as well as the short-lived reactive iodine radicals (I_2_^•−^). The stable species (I_2_/I_3_^−^ and H_2_O_2_) are mostly involved in the photokilling of gram-negative bacteria [37]. This could be explained because the thin cell wall of gram-negative bacteria allows iodine species to penetrate and kill them easier, in comparison with other microbial cells with thicker cell walls [41], while the short-lived radicals (I_2_^•−^) were most involved in the photokilling of gram-positive bacteria [37].

Some authors suggest that free iodine must reach a threshold concentration to be microbicidal and that this amount of free iodine produced is directly related to the amount of singlet oxygen produced, as well as the concentration of iodide anion present in the solution [36,37]. It is believed that due to the very short lifetime of singlet oxygen, the probability of being quenched by iodide is higher when the iodide concentration is high, thus the iodide concentration is important in aPDT [40].

When we used KI at 50 mM, a half of the usual KI concentration, in combination with EOS or RB, a strong potentiate effect in the photoinactivation of *S*. Typhimurium and *S. aureus* is still observed, compared to the PSs alone [19,20] (Figure 3; *p* < 0.05). However, it was possible to observe that when EOS at 0.50 µM with KI at 50 mM was used, an additional time of light exposure of 10 min (9.0 J/cm^2^) are needed, in comparison with the same EOS concentration with KI at 100 mM, to totally photoinactivate *S*. Typhimurium (Figure 3A). When the EOS concentration was reduced to 0.10 µM with KI at 50 mM it was not necessary an addition of light exposure to reach the total photoinactivation of *S*. Typhimurium cells, compared with KI at 100 mM. For the combination of RB at 0.25 µM and 0.50 µM with KI at 50 mM, it was observed that was necessary to use higher irradiation times to reach the total photokilling of *S*. Typhimurium cells (>7 log of reduction), compared with the KI at 100 mM (Figure 3B). The assays performed with the lowest RB concentration (0.10 µM) in the presence of KI at 50 mM showed a reduction of approximately 3 logs even after an irradiation time of 15 min (total light dose 9.0 J/cm^2^). Wen et al. [41] eradicated *E. coli* cells using KI at 25 mM and a light dose of 10 J/cm^2^, however, a RB concentration 100 times higher was used. These results suggest that if the concentration of KI is reduced, it is necessary to increase the PSs concentration or the light exposure time to achieve the same photoinactivation profile.

When EOS at 0.50 µM combined with KI at 50 mM was used against *S. aureus* it was possible to observe a similar profile, to photoinactivate *S*. Typhimurium; it was necessary to increase the time of light exposure to achieve total inhibition of the bacterium (Figure 3C). A reduction of approximately 6 log was achieved when the KI concentration was halved and it was used at the lowest EOS concentration (0.10 µM) with a light exposure of 15 min (9.0 J/cm^2^). When we tested the highest RB concentration (10 nM) in combination with KI at 50 mM, total inactivation of *S. aureus* cells was observed after 10 min of light exposure (Figure 3D). This result was the same observed for RB at 10 nM but with KI at 100 mM. It is known that the xanthene derivatives weakly binds to most microorganisms. In this case, some authors suggest that concentrations up to 100 mM of KI are necessary to have an improvement in the PS action [38,40,41]. In our study we really achieved a great improvement in the PSs’ action when KI was used at 100 mM, but for lower KI concentrations, high inactivation rates was also achieved.

### 2.3. aPDT Resistance Assays

**Figure 5:**
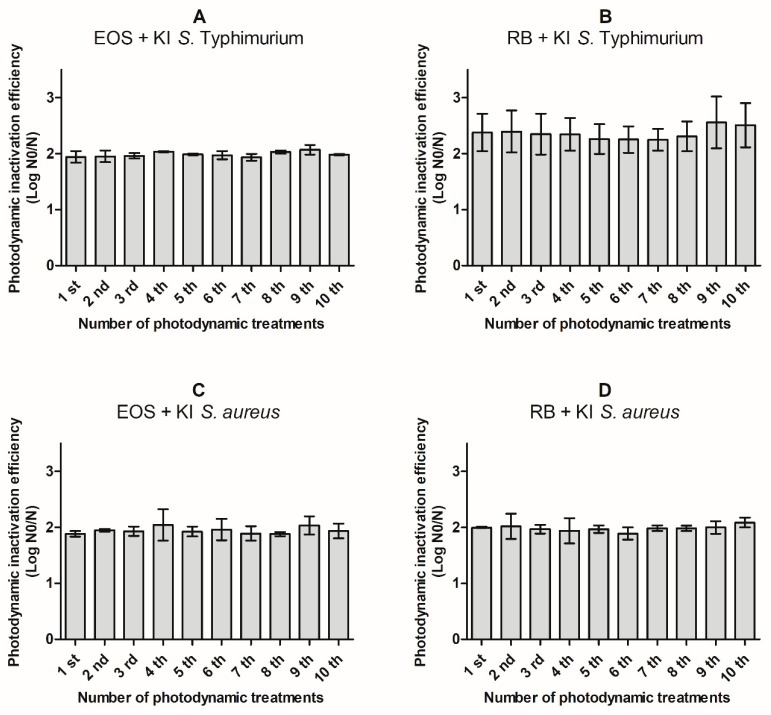
Photodynamic inactivation efficiency of ten consecutive cycles of *S*. Typhimurium (up), and *S. aureus* (down) by 0.10 µM of eosin (EOS) with 100 mM of KI (**A**,**C**), 0.10 µM of rose bengal (RB) with 100 mM of KI (**B**), and 5.0 nM of rose bengal (RB) with 100 mM of KI (**D**) after 10 min of irradiation with green LED light (530 ± 40 nm). N0 represents the plaque counts of bacterial cells before the irradiation; N represents the plaque counts after the cycle treatment; error bars indicate the standard deviation.

Actually, multidrug resistant bacteria are one of the most serious health problems in world. It is known that some multidrug resistant foodborne pathogens have been found in food for human consumption [7,10,11,12,13,14]. In this sense, aPDT can be a promising alternative once it affects a high number of microbial targets simultaneously, thus preventing the development of bacterial resistance [16,36]. In order to evaluate the potential development of bacteria resistance to aPDT treatment mediated by RB or EOS with KI, ten cycles of photoinactivation under similar conditions to the ones applied for the photoinactivation profile determination were performed. Thus, concentration of PS + KI and the irradiation time used were chosen based on the reduction of ca. 50% in the CFU levels. After each cycle of aPDT, the *S*. Typhimurium or *S. aureus* colonies, that survived to the performed photoinactivation cycle, were aseptically removed from the TSA plates and re-suspended in PBS, and then submitted to the same photoinactivation protocol. The results obtained are presented in Figure 5.

The results showed that there was no significant increase (*p* < 0.05) in resistance of *S*. Typhimurium to photosensitization after 10 consecutive sessions of 10 min with EOS or RB at 0.10 µM and KI at 100 mM (Figure 5A,B). Similar behavior was observed for *S. aureus* cells, where no significant increase (*p* < 0.05) was observed in the resistance to photodynamic action after 10 aPDT cycles (10 min) with EOS at 0.10 µM or RB at 5 nM and KI at 100 mM (Figure 5C,D). Lauro et al. [50] stated that the development of bacterial resistance could be detected by important reductions on the bacterial photoinactivation efficiency among experiments. These results clearly show the aPDT protocol with both EOS and RB with KI against *S*. Typhimurium and *S. aureus* does not induce development of resistance.

Some studies were conducted to determine if bacterial resistance occurs after several consecutive aPDT treatments [16,51]. Tavares et al. [51] also did not observe development of *E. coli* resistance by 10 cycles of 25 min of irradiation (white light 4.0 mW/cm^2^) with 5.0 µM of Tri-Py^+^-Me-PF. The same conclusions were reported by Bartolomeu et al. [16] working with three strains of *S. aureus* treated with Tetra-Py^+^-Me at 5.0 µM and illuminated by white light (4.0 mW/cm^2^) by 10 consecutive cycles of aPDT. However, to our knowledge there are no published results that determine if bacterial resistance occurs when the PS is used in combination with KI after several consecutive aPDT treatments.

## 3. Materials and Methods

### 3.1. Bacterial Strains and Culture Conditions

*Salmonella enterica* serotype Typhimurium (ATCC 14028) and *Staphylococcus aureus* (ATCC 25923) stored at −20 °C in Brain and Heart Infusion Broth (BHI, Difco, Becton Dickinson, Sparks, MD, USA) with 20% glycerol, was used in this study. The bacteria were sub cultured in Hektoen Enteric Agar (Difco, Becton Dickinson, Sparks, MD, USA) for *S*. Typhimurium and Baird Parker Agar (Difco) for *S. aureus*, and prior to experiments, they were grown overnight at 37 °C in BHI (Difco, Becton Dickinson, Sparks, MD, USA). Then, the microorganisms were harvested by centrifugation (5000× *g* for 5 min) and washed three times with 0.85% saline solution. The inoculums were adjusted to approximately 1 × 10^7^ colony-forming units (CFU) per mL and used in the experiments [20].

### 3.2. Photosensitizers and LED Light Source

A stock solution of RB and EOS (Sigma Aldrich, Darmstadt, Germany) at 1.0 mM was prepared in PBS pH 7.2, filter sterilized, standardized in a spectrophotometer (UV-Vis Beckman Coulter DU *800) and kept in the dark under refrigeration until use [19].

The green LED homemade device prototype has 252 LEDs appropriately arranged on a plate of 13 cm length × 8 cm width, with a distance from the microplate surface of 3.5 cm. The prototype has an irradiance of 10 mW/cm^2^ and a wavelength of 530 ± 40 nm. The spectral emission of the LEDs system was obtained using a spectrofluorimeter (Varian Cary Eclipse, San Diego, CA, USA). The absolute irradiance of the LEDs was evaluated with a Spectroradiometer USB2000+RAD (Ocean Optics, Winter Park, FL, USA).

### 3.3. Photostability Assay

The photostability of EOS and RB was evaluated in PBS. The samples were continuously illuminated by a set of LEDs (10 mW/cm^2^ and a wavelength of 530 ± 40 nm) for a period of 0, 5, 10 and 15 min and the LED system were adapted to the Varian Cary-60 spectrophotometer. This spectrophotometer works with phase-modulated radiation, allowing the experiment to be conducted without interference from external radiation. So, 2.0 mL of the aqueous solution containing the dyes were added in a quartz cuvette (1.0 cm optical pathway). The LED system was positioned at the top of the cuvette and the spectral reading was initiated using the kinetic method of the equipment. Finally, the spectral variations were properly evaluated [46].

### 3.4. Photodynamic Inactivation Assays

The photoinactivation assays were performed according to Silva et al. [20]. In a 24-well plate 500 µL of bacterial suspension with different concentrations of RB or EOS with KI were kept in the dark for 10 min to promote the PS + KI binding to bacterial cells before irradiation. Simultaneously, four control groups were also evaluated: positive control (C), containing only the bacterial inoculum in PBS without illumination; light control (LC), containing only the bacterial inoculum in PBS exposed to the same light conditions as the samples; KI control (KIC), containing the bacterial inoculum in PBS + KI exposed to the same light protocols and; dark control (DC) containing the inoculum and PS + KI without illumination. After incubation, the samples, LC and KIC were exposed to the green LED light for 5, 10 and 15 min.

Finally, samples from each well were serially diluted in 0.85% saline solution and plated in duplicate onto Tryptic Soy Agar (TSA, Difco, Becton Dickinson, Sparks, MD, USA). The plates were incubated at 37 °C for 24 h and the CFU/mL was counted. Experiments were carried out in duplicate and repeated three times in independent experiments.

### 3.5. aPDT Resistance Assays

In order to verify the development of resistance to aPDT treatment with RB with KI and EOS with KI, ten cycles of photoinactivation under similar conditions were performed. The concentration of PS + KI and the irradiation time used were chosen based on the reduction of ca. ~50% in the CFU levels. After each cycle of aPDT, the *S*. Typhimurium or *S. aureus* colonies, that survived to the previous cycle of photoinactivation, were aseptically removed from the TSA plates and re-suspended in PBS, and then underwent the same photoinactivation protocol. The optical density of both bacteria suspension, before each assay, was measured to prevent differences in the aPDT efficiency. The aPDT efficiency was expressed as log N0/N, where N0 and N represent the colony counts before and after the irradiation, respectively. Three independent assays in duplicate were performed [16].

### 3.6. Statistical Analysis

Statistical analysis was performed by using one-way ANOVA and the Tukey multiple comparison test (GraphPad Prism 7.0). The level of statistical significance was set at *p* < 0.05. All experiments were carried out in duplicate and repeated at least three times in independent experiments.

## 4. Conclusions

The present study demonstrated that addition of KI at both concentrations tested (50 mM and 100 mM) can strongly potentiate the aPDT mediated by the xanthene derivatives EOS and RB. The use of KI allowed a drastic reduction of the PSs concentration (at least 500 times) and promoted the inactivation even of the gram-negative bacterium *S*. Typhimurium, a bacterium which is not so prone to inactivation with xanthene dyes when used alone. It was also confirmed that *S*. Typhimurium and *S. aureus* did not develop resistance mechanisms when submitted to consecutive cycles of aPDT protocol in the presence of EOS and RB with KI. Therefore, the effective inactivation of both bacteria without development of resistance, the low price of the xanthene dyes, the nontoxic nature of KI and the possibility of greatly reducing the EOS and RB concentrations allow the development of a very promising alternative to control foodborne pathogens, forecasting its ease of potential transposition to the food industry.
